# A Review of Subsidence Monitoring Techniques in Offshore Environments

**DOI:** 10.3390/s24134164

**Published:** 2024-06-26

**Authors:** Frank Thomas, Franz A. Livio, Francesca Ferrario, Marco Pizza, Rick Chalaturnyk

**Affiliations:** 1 Department of Science and High Technology, Insubria University, 22100 Como, Italy; franz.livio@uninsubria.it (F.A.L.); francesca.ferrario@uninsubria.it (F.F.); m.pizza@uninsubria.it (M.P.); 2 Department of Civil and Environmental Engineering, University of Alberta, Edmonton, AB T6G 2W2, Canada; rc11@ualberta.ca

**Keywords:** subsidence monitoring, offshore, structural integrity, uplift, seafloor deformation, integrated monitoring approaches

## Abstract

In view of the ever-increasing global energy demands and the imperative for sustainability in extraction methods, this article surveys subsidence monitoring systems applied to oil and gas fields located in offshore areas. Subsidence is an issue that can harm infrastructure, whether onshore or especially offshore, so it must be carefully monitored to ensure safety and prevent potential environmental damage. A comprehensive review of major monitoring technologies used offshore is still lacking; here, we address this gap by evaluating several techniques, including InSAR, GNSSs, hydrostatic leveling, and fiber optic cables, among others. Their accuracy, applicability, and limitations within offshore operations have also been assessed. Based on an extensive literature review of more than 60 published papers and technical reports, we have found that no single method works best for all settings; instead, a combination of different monitoring approaches is more likely to provide a reliable subsidence assessment. We also present selected case histories to document the results achieved using integrated monitoring studies. With the emerging offshore energy industry, combining GNSSs, InSAR, and other subsidence monitoring technologies offers a pathway to achieving precision in the assessment of offshore infrastructural stability, thus underpinning the sustainability and safety of offshore oil and gas operations. Reliable and comprehensive subsidence monitoring systems are essential for safety, to protect the environment, and ensure the sustainable exploitation of hydrocarbon resources.

## 1. Introduction

Offshore oil and gas extraction and injection play a crucial role, particularly in 2024, amid the escalating global energy demand precipitated by the ongoing energy crisis. The energy transition imperative has hindered the replenishment of energy reserves, thereby exerting profound ramifications across various sectors [1,2,3,4]. Diverse industries, including transportation, heavy manufacturing, petrochemicals, heating, and electricity generation, remain reliant on hydrocarbons due to their indispensable nature in fulfilling energy requirements [3,5]. While renewable energy endeavors are indispensable, the energy needs of certain sectors necessitate the continued utilization of hydrocarbons. Furthermore, the profound economic contributions of the oil and gas sector to both global and developing economies are noteworthy, manifested through job creation, governmental revenue generation, and other socio-economic benefits [6]. Notably, the established infrastructural framework and insights garnered from exploration hold substantial significance for the energy transition, particularly in domains like Carbon Sequestration, Geothermal Energy, Underground Gas Storage (UGS), Hydrogen Storage, and subsurface applications encompassing waste management and storage [7].

**Figure 1:**
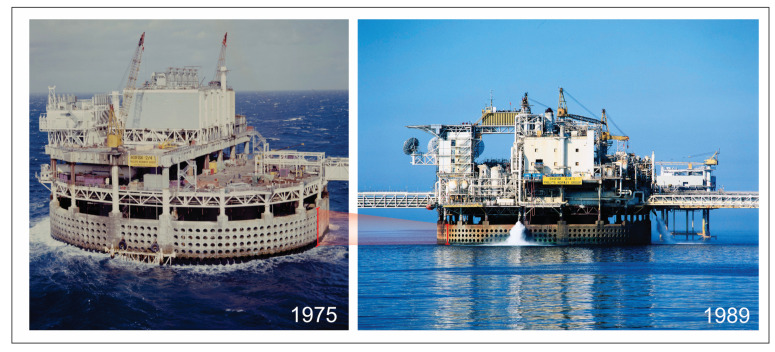
Subsidence recorded at the Ekofisk Platform over a period of 14 years: on the left, a photo taken in 1975 shows the platform shortly after its installation; on the right, the same platform in 1989 reveals significant changes due to subsidence (red marker). The picture is reproduced with permission. (Photo credits: Image 1975, ConocoPhillips/Norwegian Petroleum Museum; Image 1989, Husmo Foto/ConocoPhillips/Norwegian Petroleum Museum.

To safeguard the health of these assets and prevent unintended consequences for the activities and for the environment, subsidence monitoring is a critical component for the correct estimation, management, and mitigation of operational-related hazards [8]. Subsidence is mainly caused by the compaction of reservoirs and its transmission to the seafloor via the overburden [9,10]. Subsidence, if not regularly monitored, can cause serious damage to an infrastructure [11]. Offshore platforms, in fact, need to have a minimum clearance from the mean water level to the topside of the structure. If this air gap is reduced, it raises the risk of deck wave inundation and related wave slam; thus, the level of subsidence could have a substantial impact on a facility’s safety. Wave-in-deck loading causes (a) a higher overall platform loading and a higher danger of structural failure and (b) water getting into the cellar deck [12]. As seen in Figure 1, the loss of the air gap and the resulting reduction in platform safety during storm events led to the 1987 Ekofisk platform jacking operations and the installation of a barrier wall around the 2/4 T platform in 1989. The combined cost of these subsidence remediation efforts approached USD 1 billion [13] Additionally, subsidence can also impact productivity (there is a potential for detrimental effects caused by productivity decline), well integrity (i.e., overburden deformations may result in tubing leaks and, consequently, well workovers [10]), be a flooding hazard, damage the pipeline, and affect casing integrity [8].

Oil and gas industry management is not devoid of inherent risks. These include oil spills and catastrophic blowouts exemplified by the well-documented Deepwater Horizon incident that discharged approximately 4.9 million barrels of oil into marine environments and adversely impacted aquatic ecosystems across five states [14,15]. The prospect of induced seismicity arising from production activities [16], groundwater contamination due to factors such as methane gas migration and hydraulic fracturing [17], as well as the subsidence and uplift of the Earth’s surface [18] all represent potential hazards. In the worst circumstances, exposure to these risks can lead to the loss of human life, underscoring the critical nature of addressing and mitigating such challenges [19].

By the early detection and estimation of subsidence rates, mitigation strategies can be adopted in time, preventing catastrophic events [15]. The impact of such disasters can be severe, and remedial measures can be more difficult offshore than onshore.

Despite the considerable progress made in the monitoring of gas leakages through remote sensing techniques [11,20,21], obtaining accurate and reliable measurements of subsidence and other vertical movements of the seafloor remains a challenging issue. Therefore, a comprehensive review of subsidence monitoring techniques is necessary to ensure the safe and sustainable extraction of oil and gas from offshore fields and to mitigate the negative impact on the environment and marine life. Here, we tackle this issue, focusing on particular reference to offshore extraction or injection activities.

In this work, we will discuss the present-day available monitoring techniques, including the following: (i) hydrostatic leveling, (ii) compaction monitoring tools, such as casing collar deformation analysis, (iii) bathymetry, (iv) air gap measurements, (v) radar water-level measurements, (vi) radioactive marker technique (RMT), (vii) electric log data, (viii) tiltmeters, (ix) fiber optic cables, (x) time-lapse gravimetry and pressure, (xi) Agisco compensator, (xii) microelectromechanical systems (MEMSs), (xiii) InSAR, (xiv) GNSS time series, and (xv) bottom pressure recorder + GNSS (i.e., the MEDUSA System).

## 2. Materials and Methods

**Figure 2:**
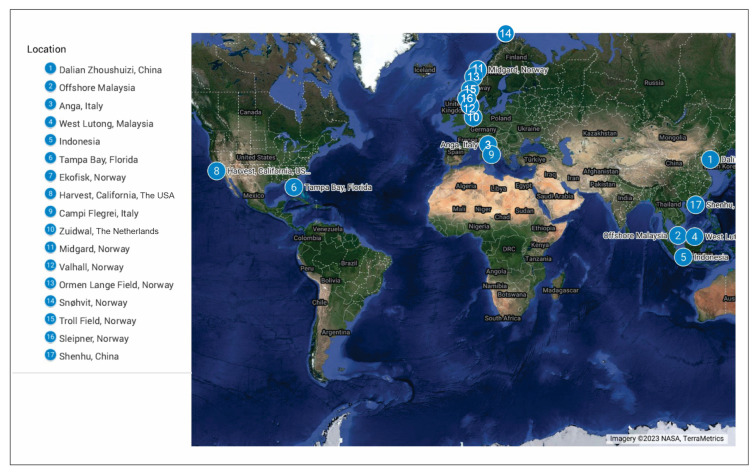
Location of the case studies analyzed in this review. The map shows the global distribution of the 17 case studies analyzed, with each numbered marker corresponding to the listed locations on the left.

The objective of this study is to comprehensively review historical and contemporary offshore subsidence monitoring techniques through a systematic literature review. This compilation encompasses techniques with accompanying case studies Figure 2, some of which may lack published data. A comparative analysis was conducted, juxtaposing data derived from the multiple techniques employed to monitor subsidence and compaction. All the data presented in this paper originate from a review of 93 papers (Table 1) published in scholarly journals.

The underlying theory governing vertical movements primarily stems from the injection or extraction of fluids/gases, directly leading to the compaction or expansion of reservoirs. This phenomenon is directly proportional to the depth of the reservoir where fluid or gas is injected or extracted. The lithological characteristics of both the overburden and underburden layers exert a pivotal influence on the extent of subsidence. Consequently, it is important to note that the compaction magnitude is not synonymous with the subsidence amount. Herwanger [22] illustrates that a reduction in the reservoir thickness during compaction induces stretching in the overburden. A rigid underburden impedes underburden rebound, whereby for a stiff underburden, a considerable proportion of reservoir compaction is translated into a downward overburden movement. Conversely, a soft underburden results in both a downward overburden movement and upward underburden movement. Notably, the Young’s modulus of a stiff underburden significantly exceeds that of a soft underburden.

In tandem with the role played by the overburden and underburden in the subsidence magnitude, the lateral extent, thickness, and depth of the reservoir equally contribute. For reservoirs at substantial depths, compaction is distributed proportionally between overburden subsidence and underburden rebound. Interestingly, for shallow reservoirs, surface subsidence predominantly accommodates compaction. This observation can be attributed to diminished overburden stretching owing to a comparatively smaller overburden. Wider reservoirs provoke more pronounced surface subsidence in contrast to narrower reservoirs with an identical volume and depth. Additionally, for infinitely extended reservoirs, uniform compaction and overburden subsidence occur without causing stress changes in the overburden [22].

As a general guideline, thicker reservoirs exhibit greater compaction in contrast to thinner reservoirs. Deep and narrow reservoirs yield limited surface subsidence, while shallow and wide reservoirs manifest considerable compaction as surface subsidence, as expounded by [22].

To reduce the impacts of compaction and enhance pore pressure dynamics, we inject fluids into the ground. This strategic intervention serves a dual purpose: it reduces the effects of compaction while boosting the productivity of the reservoir [23]. 

**Table 1:** Subsidence monitoring techniques and list of the relevant papers analyzed in this review.

Technique	Section	Analyzed References
Direct measurement techniques	Section 3.1	
Hydrostatic leveling	Section 3.1.1	[24,25,26,27]
Casing collar deformation analysis	Section 3.1.2	[28,29]
Hydrographic techniques	Section 3.2	
Bathymetry	Section 3.2.1	[30,31,32,33]
Air gap measurements	Section 3.2.2	[33,34,35]
Radar water-level measurements	Section 3.2.3	[33,36]
Radioactive marker technique (RMT)	Section 3.3	[8,37,38,39,40]
Well logging	Section 3.4	
Electric log data	Section 3.4.1	[41]
Formation–compaction monitoring tool (FCMT)	Section 3.4.2	[42]
Tiltmeters	Section 3.5	[43,44,45,46,47]
Fiber optic cables	Section 3.6	[48]
Fugro-proposed tools	Section 3.6.1	[43]
Fiber Bragg grating (FBG) strain sensor	Section 3.6.2	[43,49]
Time-lapse gravimetry and pressure	Section 3.7	[43,50,51,52,53,54,55,56]
Agisco compensator	Section 3.8	[43]
Microelectromechanical systems (MEMSs)	Section 3.9	[57,58,59]
Remote sensing	Section 3.10	
InSAR (interferometric synthetic aperture RADAR)	Section 3.10.1	[20,60,61,62,63,64,65,66,67,68,69]
GNSS (global navigation satellite system) time series	Section 3.10.2	[11,20,70,71,72,73,74,75,76,77,78]
GNSS on an anchored spar buoy	GNSS on an Anchored Spar Buoy	[79]
Bottom pressure recorder + GNSS (MEDUSA System)	Bottom Pressure Recorder + GNSS (MEDUSA System)	[80,81,82,83]

## 3. Offshore Subsidence Monitoring Systems

### 3.1. Direct Measurement Techniques

#### 3.1.1. Hydrostatic Leveling

Hydrostatic leveling utilizes a configuration characterized by two or more water-filled chambers positioned at different elevations, each equipped with a pressure sensor. The role of these pressure sensors is to gauge the hydrostatic pressure, a parameter directly related to the elevation difference between these chambers [24]. This technique is implemented through interconnected vessels, where the measurement of the water levels at both ends of an elongated tube permits the transfer of height measurements. The tubes are strategically positioned within bodies of water to facilitate accurate measurements. As seen in the Netherlands, the Rijkswaterstaat (Directorate General for Public Works and Water Management) used a specialized vessel furnished with tubes of variable lengths that could be interlinked to achieve a cumulative length of up to 12 km. This vessel was employed for hydrostatic measurements within the Wadden Sea fields. Nevertheless, factors such as the vessel’s aging condition, alterations in the occupational safety guidelines, and the emergence of global navigation satellite systems (GNSSs) for elevation determinations prompted the ship’s retirement in 2003. Consequently, GNSS measurements replaced hydrostatic measurements in the Netherlands. It is noteworthy, however, that the historical dataset obtained through hydrostatic leveling remains valuable for deformation analysis [25,26]. The subsidence within the Wadden Sea region was initially assessed utilizing leveling techniques and subsequently transitioned to GPS measurements. The case study presented by [27] exemplifies the application of precise leveling at the Zuidwal platform spanning from 2000 to 2005. This endeavor yielded a discernible subsidence rate of 1.16 mm/yr.

#### 3.1.2. Casing Collar Deformation Analysis

This approach to quantifying reservoir compaction involved measuring changes in the inter-casing distance, specifically the spacing between casing collars. These measurements, with a precision of 12 mm per casing joint [28,29], utilized a comprehensive casing collar logging system. This system integrated a specialized magnetic collar locator operated via a single conductor cable at a logging velocity of 3.7 m per minute, with the depth accurately determined through a precision vernier cable odometer. However, the effectiveness of this method depended heavily on the interaction between the reservoir rock and the casing. In instances where the casing was securely cemented in its position, the technique provided a reasonable approximation of the actual reservoir compaction. However, scenarios where the cement bond was compromised or altered due to compaction resulted in a diminished correlation between casing collar outcomes and authentic reservoir compaction. To address the challenge of mitigating uncertainty stemming from the interplay between reservoir compaction and casing collar movement, the development of a compaction monitoring tool was undertaken. While varying iterations of this tool currently exist, the fundamental approach remains uniform [8].

### 3.2. Hydrographic Techniques

#### 3.2.1. Bathymetry

**Figure 3:**
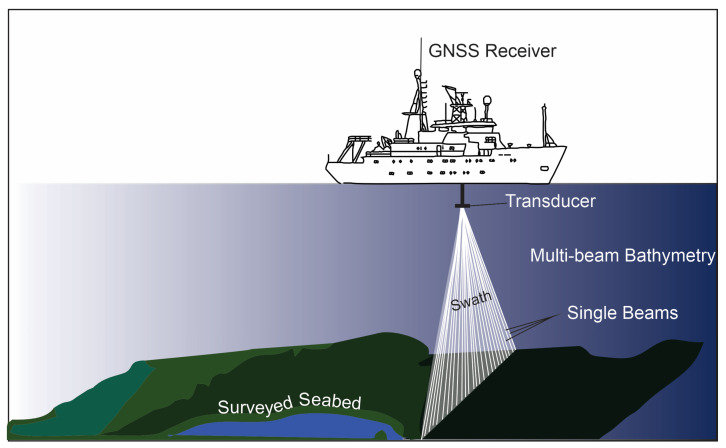
Multi-beam bathymetry survey (modified after Tay et al., 2013 [85]). This schematic illustrates the process of a multi-beam bathymetry survey conducted from a survey vessel equipped with a GNSS receiver and transducer. The transducer emits multiple beams that cover a swath of the seabed, providing detailed topographic data.

Bathymetric survey techniques have been integral to marine scientific endeavors since the early 19th century. Time-lapse single-beam and multi-beam bathymetric surveys, as seen in Figure 3, are leveraged to evaluate the magnitude and velocity of vertical movements and basin floor morphological changes [84].

In the Danish sector of the North Sea, subsidence monitoring has traditionally relied on GNSS stations installed on surface platforms. However, this approach provides single-point measurements and does not capture the full extent of subsidence in areas with long horizontal wells. To address this limitation and gain a more comprehensive understanding of regional subsidence and its potential implications for reservoir geomechanics, 4D seismic surveys were conducted. These surveys also included single-beam echo sounder data, typically underutilized for bathymetry modeling due to limited accuracy and resolution. In this context, a dedicated processing workflow was developed, significantly enhancing the accuracy of 3D bathymetric models. This integration of bathymetric data with reservoir and overburden geomechanical models enabled the calculation of seabed subsidence with an estimated accuracy of 0.3 m. This approach proved cost effective and valuable for subsidence monitoring in areas lacking 3D seabed subsidence data [30].

The single-beam echo sounder technique stands as the standard, low-risk, and conventional means of seafloor data collection. Employing a transducer, this technique projects an acoustic pulse through the water column and subsequently measures the returned signal. Typically affixed to a vessel’s hull, the accuracy and quality of the data depend on complementary technologies such as positioning and vessel attitude determination [31].

Conversely, the multi-beam echo sounder (MBES) system constitutes a sophisticated tool for contemporary marine surveys, comprising an intricate assemblage of multiple sensors. Integrating acoustic reflection, scattering, and interferometric principles, this system generates swath-style bathymetric data through numerous high-density soundings, narrow measurement beams, advanced bottom-detection algorithms, and sound velocity correction techniques. These components ensure heightened system detection accuracy and precise point coordinate reduction [32]. MBES systems are broadly categorized into two groups: beamforming MBES, which utilizes beamforming control technology (often referred to as traditional MBES), and interferometric MBES, which employs interferometry technology (also known as phase-differencing MBES or bathymetric side scan [32].

#### 3.2.2. Air Gap Measurements

**Figure 4:**
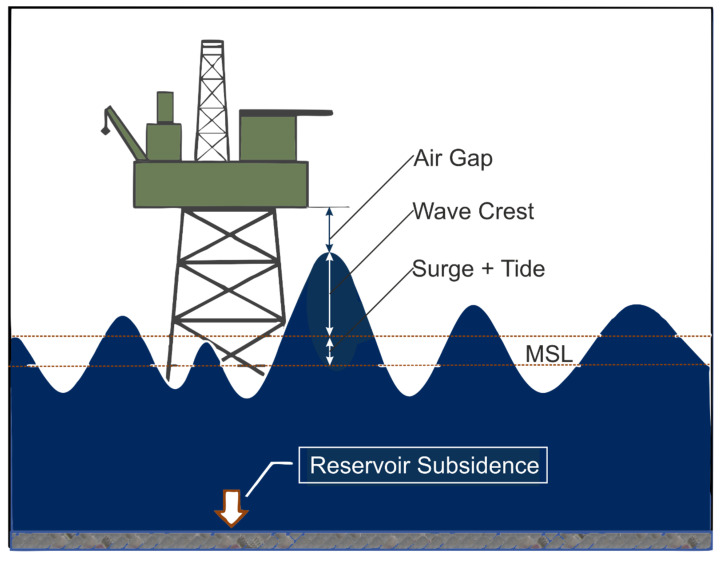
Conceptual model of water level components influencing the air gap. MSL is the mean sea level. The extreme water level comprises the wave crest + tide + SLAs (sea level anomalies) + non-tidal residuals (NTRs). An NTR is the difference between the observed water levels and the levels predicted by tidal models (modified after Anokhin and Ewans, 2018 [34]).

Air Gap measurements as seen in Figure 4 utilize the precise quantification of the vertical separation or space existing between the water surface and a stationary structure, such as a platform. Notably, SAAB WaveRadar REX systems (formerly developed by SAAB™, it was subsequently developed and marketed by RS Aqua™ to WaveRadar REX^2^ (https://rsaqua.co.uk/product/waveradar-rex2/, accessed on 2 February 2024.)) have gained extensive usage in the offshore oil and gas sector. These measurements serve as a means to deduce vertical movements of platforms, which can be attributed to both reservoir subsidence and vertical land shifts [34,35].

Previous studies on air gap surveys have shown that it is possible to monitor water levels with great accuracy, with an uncertainty of about 0.05 m [33].

#### 3.2.3. Radar Water-Level Measurements

**Figure 5:**
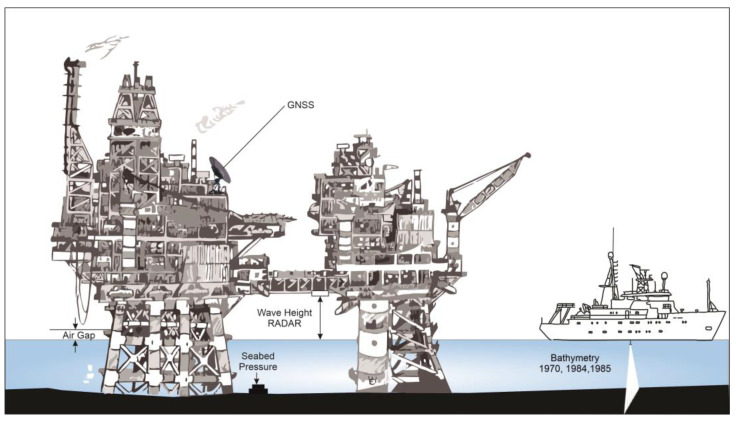
Radar water-level measurements were conducted on the Ekofisk field along with other techniques used to measure subsidence. The radar is installed on the bridge between the two platforms (modified after Rentsch and Mes, 1988 [33]).

Radar water-level measurements use the application of radar technology to ascertain the water level within a designated body of water. This methodology as seen in Figure 5 relies on radar waves transmitted from a sensor, which reflect off the water surface and return to the sensor. By accurately measuring the time taken for these radar waves to travel to the water surface and back, the distance between the sensor and the water surface is calculated. This distance directly corresponds to the water level at the exact location of the sensor [36].

Illustratively, within the context of the Ekofisk field during the temporal span, spanning from 1980 to 1984, a Plessey radar device was installed on the bridge between two platforms. The device produces an electrical potential proportional to the distance from the water surface to the device. A subsidence rate of 500 mm/yr was observed. Notably, this determination was obtained within a confidence bound characterized by individual measurements, exhibiting a precision level of approximately 200 mm. This instance underscores the effectiveness of radar water-level measurements in deciphering aquatic dynamics and quantifying changes in water levels over distinct temporal intervals [33].

### 3.3. Radioactive Marker Technique (RMT)

**Figure 6:**
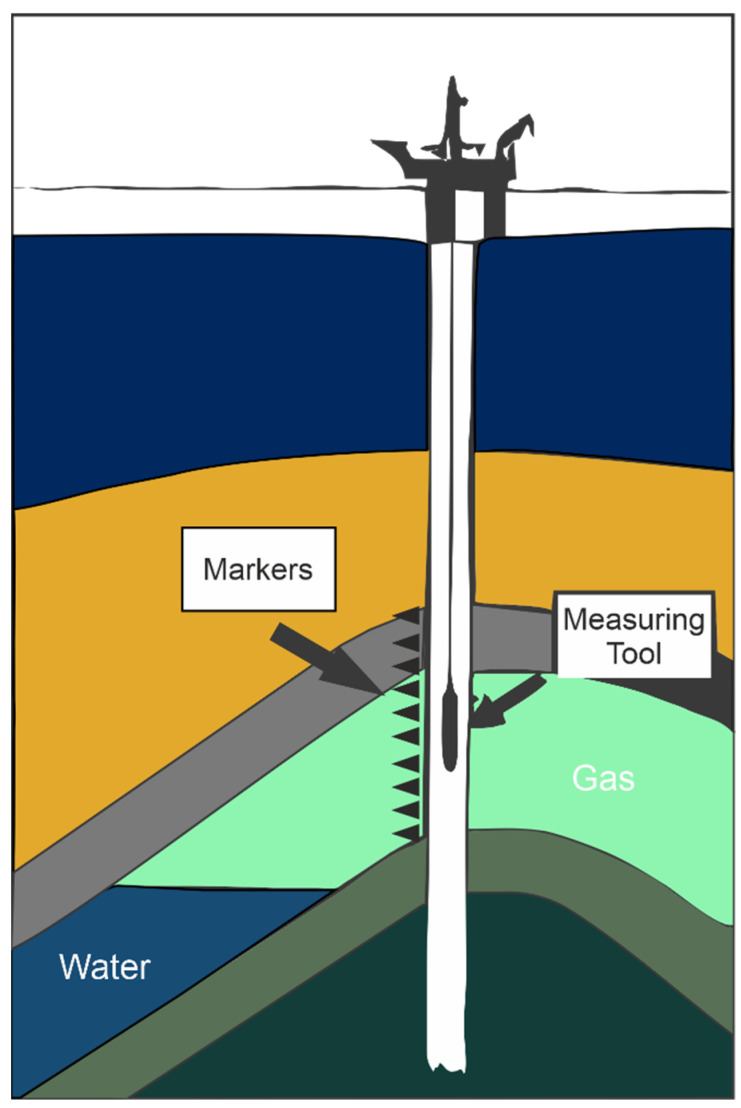
Conceptual model of the radioactive marker technique (RMT). Radioactive bullets are shot into the producing formation, followed by periodic measurements of gamma radiation (GR) to monitor alterations in the distances between these markers (modified after Macini and Mesini, 2002 [40]).

The radioactive marker technique (RMT) involves introducing radioactive projectiles, often using 137Cs due to its suitable half-life, into the geological formation before casing installation (Figure 6). These projectiles are fired at regular intervals, often in increments of 10 m, using a modified perforating gun. To gauge compaction, a wireline tool equipped with multiple gamma ray detector units is maneuvered across the interval containing the radioactive projectiles. Employing statistical analysis of the data and incorporating accelerometer corrections to account for tool movement irregularities, alterations in the distance between two projectiles can be precisely measured down to a resolution of one centimeter [8,37].

Thus, the primary objectives of deploying the RMT in productive fields involves two key aspects: (i) a direct measurement of reservoir compaction linked to hydrocarbon extraction activities and (ii) an estimation of the mechanical properties of the reservoir rock to enhance the accuracy of subsidence modeling predictions [38].

Although the RMT had modest beginnings within the context of the Champion oil field offshore Brunei, its subsequent adoption extended to significant fields such as the Groningen gas field in the Netherlands and fields characterized by considerable subsidence like the Ekofisk field in Norway, as documented by [41]. Notably, its applicability also expanded to the Gulf of Mexico [37,39,40]

In the realm of the Italian Adriatic offshore, the scenario evolved due to the authorization granted by the Italian ministry for the development of offshore gas reservoirs situated in proximity to the coastline. Given the geographical proximity to the coastline, the companies operating in this area are forced to undertake both environmental risk assessments and subsidence monitoring (art. 6.a.1, paragraph 4 of law 349/1986). It is significant to note that a regulatory law has been enacted, mandating the RMT as the primary technique for subsidence monitoring. This legislative framework carries significant weight, as it underscores the recognition of RMT measurements from a judicial perspective [40].

Challenges associated with RMT implementation refer to (i) data collection within the in-situ environment and (ii) the subsequent interpretation and utilization of the collected data [38].

### 3.4. Well Logging

#### 3.4.1. Electric Log Data

Electric log data provide a precise and detailed record of the geological formations encountered within a borehole. This technique involves lowering specialized tools into a well to continuously measure various physical properties of the rocks and fluids. Electric log data allow geoscientists to detect changes over time in the earth’s strata that could indicate compaction or uplift.

This geophysical method is particularly adept at effective subsidence, because it captures the in-situ condition of subsurface strata, enabling the identification of layers undergoing compaction. By regularly collecting electric log data at different time intervals, it is possible to observe the subsidence progression and infer the mechanical properties of the reservoir rocks.

To monitor subsidence at the Ekofisk field, electric log data played a crucial role in determining the compaction rates of distinct underground layers. Advanced logging tools include the compensated neutron log (CNL), thermal decay time (TDT), borehole compensated sonic (BHCS), and neutron detector log (NDL) methods. The outcomes yielded noteworthy figures of compaction, with calculated rates of 325.12 mm/yr, 304.8 mm/yr, 192.5 mm/yr, and 762 mm/yr, respectively, as derived from each respective technique [41].

The discrepancies in the observed magnitudes of compaction can be attributed to several factors, including variations in the measurement times, the distinct wells chosen for investigation, and, notably, intermittent halts in production activities [70]. These measurements were conducted from 1982 up until January 1985, thereby offering a comprehensive perspective on the evolution of subsidence patterns within the Ekofisk field [41].

#### 3.4.2. Formation–Compaction Monitoring Tool (FCMT)

The formation–compaction monitoring tool (FCMT) constitutes a specialized apparatus designed for the detection and quantification of formation–compaction dynamics within subsurface reservoirs. Utilizing advanced wireline technology, this tool integrates an array of precision sensors that are strategically positioned to facilitate continuous measurement and monitoring of changes in formation properties, particularly those related to compaction and subsidence phenomena [42].

The apparatus comprises four gamma-ray detectors used to ascertain the spatial coordinates of radioactive markers. It accurately measures the separation distance existing between these markers, with a precision reaching 2.54 mm over a span of 30 feet. The FCMT’s operational principle involves deploying the tool into the wellbore, where it collects real-time data on variations in the formation dimensions and mechanical behavior. These measurements provide valuable insights into the temporal evolution of subsurface compaction processes, offering a comprehensive understanding of how reservoirs respond to hydrocarbon extraction and other subsurface activities [42].

Although this technique is quite like the RMT, the FCMT provides continuous, real-time, high-precision measurements within the wellbore, whereas the RMT offers periodic, interval-based monitoring using radioactive markers placed in the formation. The choice between the two techniques depends on the specific requirements of the monitoring program, such as the desired precision, frequency of measurements, and the type of subsurface data needed.

### 3.5. Tiltmeters

Tiltmeters are sensitive instruments used to measure very small changes in inclination relative to gravity along two perpendicular axes. They function by observing the position of a gas bubble in a liquid within a sealed glass sensor, with precision electronics detecting minute resistivity changes as the bubble moves when the device tilts. Modern high-resolution tiltmeters, such as those developed in collaboration between Pinnacle Technologies™ and Lawrence Livermore National Laboratory™, can detect tilts as small as one nanoradian, an angle that equates to one part in a billion. Tiltmeters primarily measure the rate of elevation change, which is easier to determine accurately than the absolute elevation. They can be installed downhole to pinpoint the specific depths where the subsurface is compacting or expanding, according to [86]

**Figure 7:**
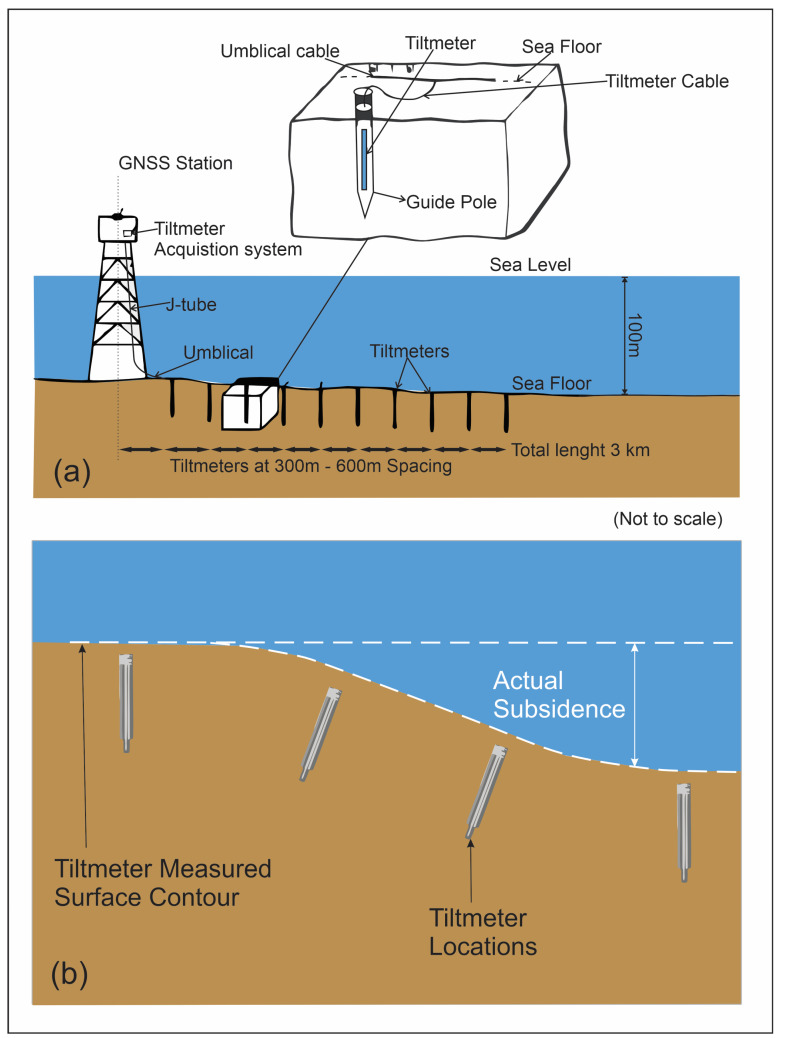
(**a**) Tiltmeter system layout illustrating the components and setup for monitoring ground tilt (modified after Miandro et al., 2015 [54]); (**b**) graphical depiction of a tiltmeter array configuration designed to detect subsidence-induced slopes, showing how multiple tiltmeters are positioned to measure changes in ground inclination (modified after Wolhart et al., 2005 [80]).

As explained in the account by [43], the company D’Appolonia™ proposed adaptations to a preceding study conducted by Anderson [44], Fabian and Villinger [45] with the tiltmeter. The modifications included the utilization of tiltmeters for analyzing surface deformations linked to volcanic activities and seismicity. This technique, as endorsed by Miandro et al. and Temizel et al. [43,46], emerges as an appealing methodology for assessing alterations at the ground level, with the collection of surface tiltmeter data in conjunction with GNSS measurements. The proposed system’s architecture as seen in Figure 7 incorporates pivotal components such as a GNSS station for capturing vertical platform displacement, a J-tube for umbilical purposes, an array of tiltmeters systematically positioned on the seafloor with predefined orientations and spacing, and an umbilical cable encompassing electrical conduits and robust cordage, serving to connect the tiltmeters to the data acquisition system established on the platform. Detailed design specifications for key system constituents, including the tiltmeter, guide pile for tiltmeter installation, tiltmeter cable, and umbilical cable, were also furnished.

Two primary categories of tiltmeters exist, specifically short-baseline tiltmeters (SBTs) and long-baseline tiltmeters (LBTs). SBTs feature a baseline of about less than 1 m, offering advantages in cost effectiveness and installation convenience. However, SBTs are predominantly sensitive to short-wavelength tilt variations and inherent noise. In contrast, LBTs, boasting a baseline exceeding 10 m, present several potential merits over the SBT design. Their extended baseline mitigates susceptibility to short-wavelength noise, thereby catering to broader-scale deformation patterns. Notwithstanding, the implementation of LBTs tends to be more financially demanding and operationally intricate compared to SBTs, as noted by [44].

Key advantages of surface tiltmeters include the following: (i) real-time monitoring of subsidence and uplift events attributed to production and injection activities; (ii) identification of areas susceptible to wellbore instability; (iii) determination of optimal production and injection strategies to avert subsidence and uplift occurrences; (iv) detection of geological faults and natural fractures; (v) determination of hydraulic fracture occurrences and orientations; (vi) calibration of reservoir simulation models [47].

### 3.6. Fiber Optic Cables

Fiber optic cables are based on the principle of observing changes in the light signal transmitted through the cables. These modifications can occur due to alterations in the physical characteristics of the material surrounding the wire, such as temperature, pressure, or strain, that change the light’s phase, intensity, or polarization. More specifically, DTS (Distributed Temperature Sensing [87]), DAS (Distributed Acoustic Sensing [88]), and DSS (Distributed Strain Sensing [89]) technologies make use of a fiber optic cable as a continuous sensor to measure the temperature, acoustics, or strain across its length.

#### 3.6.1. Fugro-Proposed Tools

**Figure 8:**
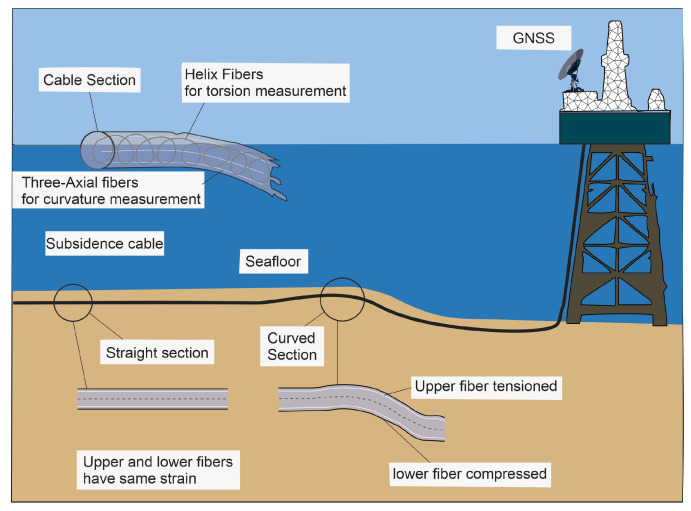
This diagram illustrates the method of measuring subsidence through strain measurement. Strain sensors are strategically placed to detect minute deformations in the ground, which are indicative of subsidence. The layout and components of the system are adapted from Miandro et al., 2015 [43], showing how strain measurement can provide precise data on subsidence rates and patterns.

In 2010, Fugro Geoservices B.V. proposed fiber-optic based tools for subsidence analysis, as described in detail by [43]. Fugro developed three distinct methodologies reliant on fiber optic technology for subsidence measurement purposes:

- Strain-based cable shape determination: A pivotal facet of subsea cable deployment involves the arrangement of cylindrical cables (Figure 8) within trenches on the seabed. Ensuring the cable’s capacity for torque during installation is essential. Accurate strain measurements are crucial for precisely assessing and documenting this torque phenomenon.
- Cable inclination measurement: In scenarios where specific cable sections undergo vertical displacement, the inclination of contiguous segments immediately preceding and succeeding the affected region experiences corresponding adjustments.
- Pressure measurement: The vertical movement of a cable section engenders modifications in the pressure within the particular segment relative to pressure levels observed in other cable regions.

#### 3.6.2. Fiber Bragg Grating (FBG) Strain Sensor

A specialized optical sensing apparatus known as the fiber Bragg grating (FBG) strain sensor is employed to quantify strains or deformations within structures. The operational principle of this sensor is rooted in the Bragg wavelength shift phenomenon, wherein alterations in the wavelength of light reflected by a fiber Bragg grating are induced by mechanical strains imposed on the optical fiber [49]. The FBG strain sensor is constructed with an optical fiber that has been purposefully modified by introducing a periodic modulation in its refractive index along its length. Upon introduction of light into the fiber, a specific wavelength of light is reflected due to periodic index variation. As the optical fiber undergoes mechanical strain or deformation, the grating period experiences adjustments, thereby leading to a shift in the reflected wavelength. Through the careful monitoring of this wavelength shift, the extent of strain or deformation experienced by the sensor can be precisely ascertained. These sensors offer a range of merits, including heightened precision, imperviousness to electromagnetic interferences, robustness, and the capacity to gauge strain at multiple locations along a single optical fiber [49]. 

As described by [90], due to the uncertainties surrounding GNSS technology in China at that time, FBG strain sensors were employed in their platform, which was a six-legged jacket platform situated in the northeastern region of Bohai Bay. The strain responses on the piles were monitored by installing FBG strain sensors, with some of them positioned on the pile surfaces to capture the changing forces acting on the piles. Alongside the FBG strain sensors, equipment for differential settlement monitoring was installed on six other piles to observe structural responses. FBG strain sensors were also placed on the platforms’ beams, which are sensitive to strain responses caused by subsidence. Additionally, tiltmeters were installed on the beams to monitor the angles of subsidence and assess any asymmetry in the responses.

### 3.7. Time-Lapse Gravimetry and Pressure

Considering the substantial financial implications associated with 4D seismic investigations, an endeavor to mitigate costs while comprehensively studying reservoir compaction has prompted the utilization of timelapse gravimetry in conjunction with pressure measurements (4D gravimetry). This combined approach affords an enhanced understanding of reservoir compaction, encompassing insight into reservoir properties and lateral compartmentalization. Notably, 4D gravimetry possesses sensitivity towards both reservoir compaction and alterations in density distribution throughout production processes [50]. A prevalent application involves the monitoring of gas–water contact movements [50].

Within each designated field, an array of gravity and pressure measurements is undertaken atop 20 to 120 semi-permanent concrete stations established on the seafloor, contingent upon the specific field’s dimensions. Each sensor frame encompasses three relative gravimeters and three pressure sensors. The orchestration entails sequential positioning of a survey vessel directly above the stations, while a remotely operated vehicle systematically deploys the sensor frame to conduct measurements, lasting for approximately 20 min. The station locations encompass both positions directly above the field and its periphery; the latter stations serve as temporal references in the context of time-lapse analysis. A survey’s duration varies from one to five weeks, contingent on the dimensions of the respective field. Survey sequences are meticulously planned, commencing and culminating at select base stations, strategically chosen to mitigate potential uncertainties stemming from instrumental drifts. In specific station subsets, tide gauges are deployed throughout the survey period to facilitate the correction of raw pressure measurements for tide-induced fluctuations and other oceanographic influences. These refined pressure data are subsequently converted into station depth measurements, thereby enabling the precise monitoring of subsidence phenomena with a mm scale accuracy [50].

Water pressure-based subsidence monitoring has been effectively executed across more than ten hydrocarbon-producing fields offshore Norway (an example of the same from Ekofisk field is shown in Figure 5), with certain programs extending beyond two decades. The body of scrutinized instances comprehensively encompasses Norwegian scenarios, where both gravimetry and pressure methodologies have been harnessed in tandem. Notably, the combined approach has been applied to noteworthy fields including Midgard, Valhall, Ormen Lange, Snøhvit, Troll, and Sleipner. Evident subsidence rates of 10.75 mm/yr, 250 mm/yr, 4.83 mm/yr, 1.88 mm/yr, 12.17 mm/yr, and 20.8 mm/yr have been documented in studies [51,52,53,54,55,56]. A notable advantage distinguishing this technique from the GPS and InSAR lies in its capacity for comprehensive area monitoring, thereby augmenting its breadth and practicality.

### 3.8. Agisco Compensator

Agisco™ in 2010 introduced an innovative tool tailored for the monitoring of offshore subsidence [43]. The operational principle of the Agisco™ compensator is similar to pressure gauges, which operate based on the principle that forces are generated by pressures within a column of liquid. Its mechanism relies on detecting changes in pressure within a cable that are influenced by altitude adjustments. Employing pressure transducers, it accurately measures the shifts in land or seabed levels. This tool’s innovation lies in its ability to offer real-time, precise data on subsidence by observing internal pressure variations that correspond with altitude changes.

This apparatus represents a reimagined iteration of an existing technology that has demonstrated efficacy in monitoring the horizontal bending tendencies of structures such as dams, harbors, and airport runways. The novel design necessitated a comprehensive consideration of the intricate challenges linked to underwater deployment in conjunction with a meticulous reassessment of the tool’s configuration and structural robustness. This approach aimed to ensure a reliable, secure, and fault-tolerant installation of the tool at the designated water depth of 100 m [43].

At the core of the compensator’s function is its ability to adjust to changes in the liquid’s volume within the hydraulic system. It can also handle minor shifts in the system’s height and related pressure changes effectively. Instances of altitude-independent volume changes, attributed to factors like thermal liquid expansion, shocks, or the constriction of hoses, warrant attention. Furthermore, the occurrence of “shockwaves” during installation or maintenance endeavors can induce perturbations in the circuit. The pressure modifications arising from volume variations or shockwaves hold the potential for significant magnitudes, raising concerns about potential damage to the pressure transducers. Hence, it was imperative to implement efficacious mechanisms for constraining and mitigating such pressure changes [43].

### 3.9. Microelectromechanical Systems (MEMSs)

Microelectromechanical systems (MEMS) inclinometers and accelerometers, which have witnessed significant developments in recent decades, particularly within the domain of landslide detection, measure horizontal displacement. These sensors are characterized by their compact size, low power consumption, and heightened reliability. MEMS accelerometers find utility in monitoring ground subsidence attributed to subterranean excavations during tunnel construction [57]. Their application has extended effectively to offshore subsidence monitoring as well. In comparison to conventional sensors, MEMS sensors hold distinct advantages such as diminutive dimensions, lightweight structures, minimal power consumption, cost effectiveness, robust reliability, facile integration of intelligence, and digital capabilities. The utilization of MEMS sensors has witnessed substantial adoption in both Japan and China [58].

**Figure 9:**
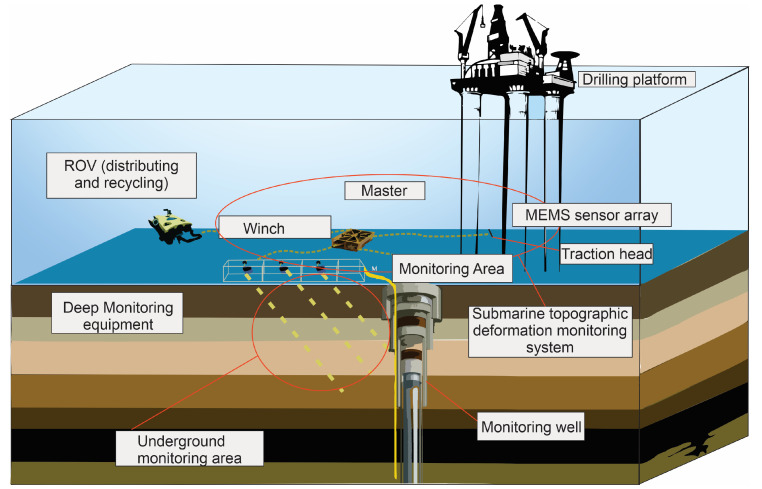
Conceptual model of MEMS sensor array in a submarine layout (modified after Chen et al., 2019 [59]). The array is designed to monitor subsidence by measuring various parameters such as pressure, tilt, and strain. The sensors are arranged strategically to cover a wide area, providing comprehensive data on subsidence patterns and rates. This model illustrates the potential for high-resolution, real-time monitoring of subsidence in underwater settings.

A terrain monitoring system founded on the MEMS sensor array is crafted by deploying multiple MEMS six-axis sensors on the seafloor surface. The arrangement as seen in Figure 9 comprises four vertically aligned arrays, each containing 21 sensors spaced at 1-m intervals in a diagonal configuration. This arrangement effectively covers a square area measuring 30 × 30 square meters.

In a specific case study as shown by Ge [58], MEMS sensors were deployed in waters with a depth of 1200 m. The experiment was conducted using the Haima-2 remotely operated vehicle (ROV) MEM for a duration of 6 months, resulting in an average maximum displacement calculation of 60.8 mm/yr. In a preceding experiment, the Haima-2 ROV underwent comprehensive pressure tests to assess the feasibility of the underwater winch and sensor array for application at a seafloor depth of 3000 m. Also, the stability of communication between the acquisition and control systems and the capacity of the MEMS sensors to withstand pressure at 3000-m depths were examined. The pressure cylinder test, conducted at 35 MPa for 18 h, revealed a maximum overall error of 2.47 mm when comparing results to those obtained under normal pressure conditions.

### 3.10. Remote Sensing

#### 3.10.1. InSAR (Interferometric Synthetic Aperture RADAR)

**Figure 10:**
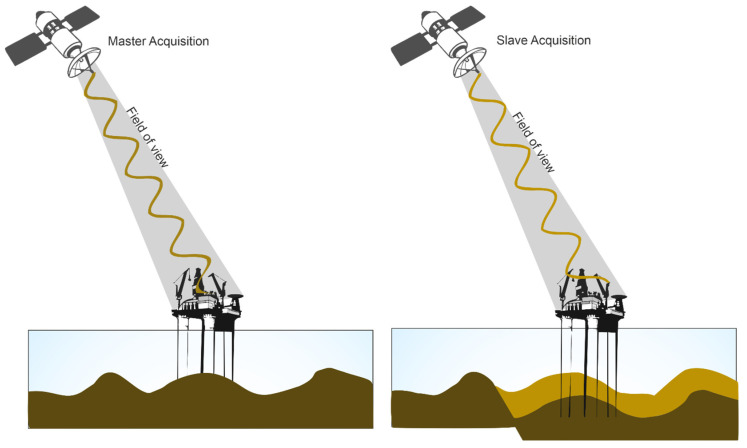
InSAR measurements from a platform (modified from https://nisar.jpl.nasa.gov/mission/get-to-know-sar/interferometry/ accessed on 28 September 2023). This figure shows InSAR (interferometric synthetic aperture RADAR) measurements captured from a satellite platform.

Interferometric synthetic aperture RADAR (InSAR) entails the utilization of two or more SAR images (Figure 10), captured over the same geographical area, to deduce the deformation components of phase change between the two passages by the subtraction of all the other possible components to phase shift (i.e., the ellipsoidal and topographic components and tropospheric delay [60,61].

In the realm of InSAR methodologies, various techniques exist, including Persistent Scatterer Interferometry (PSI), Stanford Method for Persistent Scatterers (StaMPS), Small Baseline Subset (SBAS), and SqueeSAR. PSI harnesses persistent scatterers, small objects with dimensions below the SAR resolution cell, to establish its time series algorithm [62,91]. StaMPS, sharing similarities with PSI, redefines persistent scatterers as objects with stable phase characteristics irrespective of their amplitude [63]. SBAS employs distributed scatterers and singular value decomposition to interlink independent, unwrapped interferograms temporally [64,65]. SqueeSAR, positioned as the second generation PSInSAR™ by its developer TRE [66], distinguishes itself through the amalgamation of persistent and distributed scatterers in time series analysis.

It is important to acknowledge a noteworthy limitation inherent in InSAR analysis. Primarily focusing on the platform as an isolated point, the methodology often computes the mean deformation of the entire platform. In consequence, this approach may not adequately account for potential deformation gradients along different facets, thereby constraining the accuracy and comprehensiveness of the analysis.

While InSAR is comparatively less prevalent in our case studies, instances of offshore subsidence detection using InSAR have been documented. Among these case studies, one case pertains to an artificial island in China, specifically Dalian Jinzhou Bay International Airport (DJBIA) [67]. Additionally, three other cases arise from offshore regions including Malaysia [68], Anga, Italy [20], and an undisclosed location [69]. These investigations yielded subsidence measurements of 6 mm/yr, 8.57 mm/yr, and 6.3 mm/yr, respectively.

#### 3.10.2. GNSS (Global Navigation Satellite System) Time Series

Global navigation satellite system (GNSS) technology has gathered substantial prominence in offshore subsidence monitoring, mainly owing to its intrinsic precision and dependability. GNSS technology allows us to measure subsidence rates, with a level of precision that spans a few mm/yr, as expounded upon by [71].

**Figure 11:**
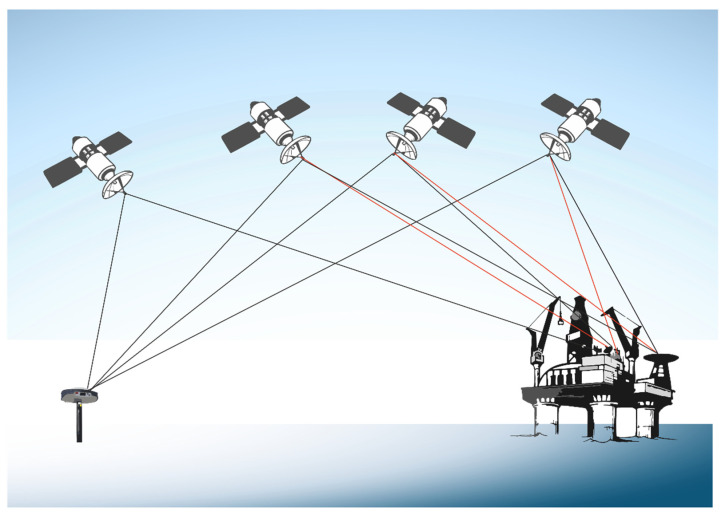
This figure illustrates the principle of monitoring platform subsidence using repeated global navigation satellite system (GNSS) GPS surveys or continuously operating GPS (CGPS) receivers. By repeatedly measuring the precise positions of points on the platform over time, subsidence can be accurately detected and quantified (modified after Andreas et al., 2018 [71]).

In this context, GNSS receivers are securely affixed to fixed infrastructural entities, including oil platforms, drilling rigs, and tethered buoys, firmly anchored to the seabed substrate. These receivers undertake the continuous accumulation of GNSS data over designated temporal intervals, relying on inputs from a minimum of four satellites (Figure 11). Subsequent data processing endeavors analyze the vertical displacement measurements of the pertinent structure and its foundational support. It is pivotal to note that the GNSS receivers are perpetually engaged in the reception of signals emitted by numerous GNSS satellites, encompassing both the navigation message and the carrier signal [71].

The GNSS framework integrates a diverse array of satellite navigation systems, including contemporary iterations of the Global Positioning System (GPS (https://www.gps.gov/ accessed on 28 September 2023)), Galileo (https://www.gsc-europa.eu/ accessed on 28 September 2023) the BeiDou Navigation Satellite System (BDS) (http://en.beidou.gov.cn/ accessed on 28 September 2023), and GLONASS (https://glonass-iac.ru/en/about_glonass/ accessed on 28 September 2023). This technological configuration empowers civilian end users to engage with these systems, engendering an integrated operational framework [11].

Amongst the spectrum of GNSS methodologies, the GPS stands as a pre-eminent and widely embraced technique in the domain of offshore subsidence monitoring [72]. Comprising a constellation of 24 satellites engaged in orbits of approximately 12 h, the GPS was conceived and realized under the auspices of the United States Department of Defense.

In the realm of GPS carrier observations, two principal methodologies hold significance: code-based and carrier-based methods [92]. The code-based approach, also known as pseudo range positioning, quantifies the time interval taken for the transmission of satellite signals from the satellite to the receiver. While this method is relatively straightforward and cost effective, its accuracy is susceptible to distortions arising from atmospheric conditions, satellite clock inaccuracies, and multipath interference [71]. In contrast, carrier-based positioning integrates the phase of the carrier wave with the code to ascertain the distance between the receiver and individual satellites. This technique offers a heightened accuracy compared to code-based methods, albeit demanding advanced equipment and intricate processing techniques. Notably utilized for dynamic object tracking, this method can achieve decimeter-level precision [73]. The GNSS data furnish a comprehensive three-dimensional displacement vector, encompassing two horizontal and one vertical component. This configuration enables not only the quantification of land subsidence but also horizontal land motion.

Standard Point Positioning (SPP) denotes a GNSS positioning methodology wherein the receiver’s position is derived exclusively from the established satellite positions. At least four satellites are requisite to solve the navigation equations encompassing four unknowns: the receiver’s position and associated errors. This technique yields several meters of accuracies [73].

Differential GPS (DGPS) stands as another salient GNSS positioning method, leveraging an array of reference stations with precisely known positions to rectify errors inherent in satellite signals. DGPS significantly augments GNSS positioning accuracy, particularly within regions characterized by limited satellite visibility or elevated interference levels [71]. This can be achieved with both Real-Time Kinematic (RTK) and Precise Point Positioning (PPP) techniques. PPP relies on a single receiver and mandates clock and orbit corrections, while RTK employs a stationary base station to transmit corrections to the mobile rover receiver. The PPP approach, conversely, utilizes precise satellite orbit and clock data to compute positions [71].

The examined case studies cover diverse geographical localities worldwide, including Anga, Italy [20]; Offshore Italy [74,75]; West Lutong [11]; Malaysia; Indonesia [71]; Tampa Bay, Florida [76]; Ekofisk, Norway [70]; and Harvest, California, USA [77,78].

Errors encountered in GNSS measurements may stem from various sources, including the influence of uplift forces from wave and wind actions. Additionally, errors can arise from satellite multipath effects, inaccuracies in satellite orbits, ionospheric distortions, and tropospheric anomalies [11].

##### GNSS on an Anchored Spar Buoy

The blend of the global navigation satellite system-acoustic (GNSS-A) technique allows us to evaluate absolute horizontal and vertical seafloor crustal deformations at the centimeter scale [79]. 

Originating in the 1980s, the vessel-based GNSS-A approach, founded on the installation of seafloor stations in advance, affords commendable precision in observations. However, this method is encumbered by physical and logistic constraints, with expenses reaching nearly USD 1000 per day and mobility capped at velocities ranging from 20 to 30 km/h [79]).

The integration of a UAV-based global navigation satellite system-acoustic (GNSS-A) emerges as an experimental avenue. This technique leverages an unmanned aerial vehicle (UAV) or sea vessel for ground motion monitoring. The interplay of GNSS observations and an attitude meter gauges the ground motion, while acoustic ranging facilitates the measurement of distances between the seabed and the vessel along designated survey lines [79]. 

However, due to economic considerations, a novel approach has been delineated in the scholarly work conducted by Yokota [79].Their research introduces the application of UAV-based GNSS-A via a floatplane UAV HAMADORI6000 prototype model, specifically engineered for sea surface takeoffs and landings. This innovation bears the dual advantages of reduced operational costs, approximately USD 100 per day, and heightened maneuverability, boasting speeds of up to 80 km/h. Such attributes expedite data acquisition and expand the survey coverage, surpassing the confines of stationary platforms like buoys or oil installations.

Comparatively, the horizontal position determinations derived from vessel-based GNSS-A data exhibit a degree of precision within the range of ±2–4 cm, as exemplified by the pinnacle observation accuracy of the current GNSS-A technology. Contrarily, the UAV-based GNSS-A real-time data manifest horizontal positioning fluctuations generally within ±35 cm [79]).

##### Bottom Pressure Recorder + GNSS (MEDUSA System)

As explained by Iannaccone et al. [80] and De Martino et al. [81], the technique herein was firstly applied in the evaluation of vertical seafloor deformations within the shallow marine sector of the Campi Flegrei caldera in southern Italy. This assessment draws from data acquired between April 2016 and July 2017 in the Gulf of Pozzuoli, facilitated by a novel marine infrastructure termed MEDUSA. Comprising four stationary buoys, each furnished with GPS receivers, MEDUSA is connected via cabling to seafloor multisensor modules housing bottom pressure recorders (BPRs).

Conceived upon the previous CUMAS concept, which hosts a broadband seismometer, a low-frequency hydrophone, and a high-precision BPR [82,83], MEDUSA entails the establishment of four marine monitoring stations. Each station comprises a buoy outfitted with a continuous GNSS receiver, linked through cabling to a subsea module incorporating an array of geophysical and oceanographic sensors. In contradistinction to conventional monitoring buoys, which remain affixed to the seabed via cabling and are subject to movements driven by water currents and sea level fluctuations, MEDUSA’s buoyancy bodies reside several meters beneath the sea level, engendering a heightened stability. This stability is realized through two distinct mechanical configurations. Among the four deployed buoys, A and C occupy a sea depth of approximately 40 m and are equipped with elongated steel poles inserted into the buoy’s body, which is then anchored to seafloor concrete ballast. Conversely, buoys B and CUMAS, positioned at depths of roughly 76 m and 96 m, respectively, necessitate an alternative approach due to impracticality of a single long pole. Consequently, a steel cable extends the pole, anchored to the concrete ballast. Functioning as semi-rigid systems coupled to the seabed, MEDUSA buoys offer a stable platform for seafloor geodetic measurements. Perturbations of the seafloor translate to detectable movements in the buoy’s visible section, measurable via the installed GNSS station [80].

Moreover, the buoy features an incorporated cable for power provision, data transmission, and GPS clock synchronization with the seafloor module. This module accommodates an assemblage of geophysical and oceanographic sensors, encompassing a three-component broadband seismometer, a state-of-the-art triaxial microelectromechanical system (MEMS) accelerometer, a low-frequency hydrophone, and a high-resolution BPR employing quartz technology. The buoy’s power source comprises rechargeable batteries coupled to solar panels. The data flow encompassing scientific and status sensor readings from the MEDUSA system is conveyed in a real-time and continuous fashion through a 5.0 GHz radio link to the INGV-OV Monitoring Center in Naples, subsequently harmonized with data originating from permanent land networks. Employing this methodology, the researchers effectively quantified a subsidence rate of 33.6 mm/yr between April 2016 and July 2017 [80]. De Martino et al. [81], in a survey conducted from July 2017 to May 2020, observed that there were higher vertical displacements of up to 61.7 mm/yr, with an error of 1.3 mm/yr.

## 4. Case Studies

The following sections delve into specific case studies that illustrate the application and effectiveness of various subsidence monitoring technologies. These studies highlight the utility of different methodologies in understanding subsidence phenomena within offshore oil fields, focusing on the Ekofisk, Valhall fields in Norway, and Anga in Italy. Each case study sheds light on the geographical context, reservoir characteristics, operational activities, and the impact of these factors on subsidence rates.

### 4.1. Ekofisk Field, Norway

Located in the North Sea around 320 km offshore from Stavanger, Norway, the Ekofisk field is well known for having played a significant part in the adoption of various subsidence monitoring techniques. This field, which is located at a depth of approximately 3000 m, contains the main producing horizons of the Tor and Ekofisk formations. These formations are characterized by Danian and Maastrichtian age fractured chalk layers, with porosities up to 50%. These reservoirs begin at a subsea level of roughly −2957 m and have a combined thickness of about 305 m. Because of continuous extraction operations, the formation pressure, which was formerly around 48,935 KPa, has now decreased to approximately 27,579 KPa [33,41].

**Figure 12:**
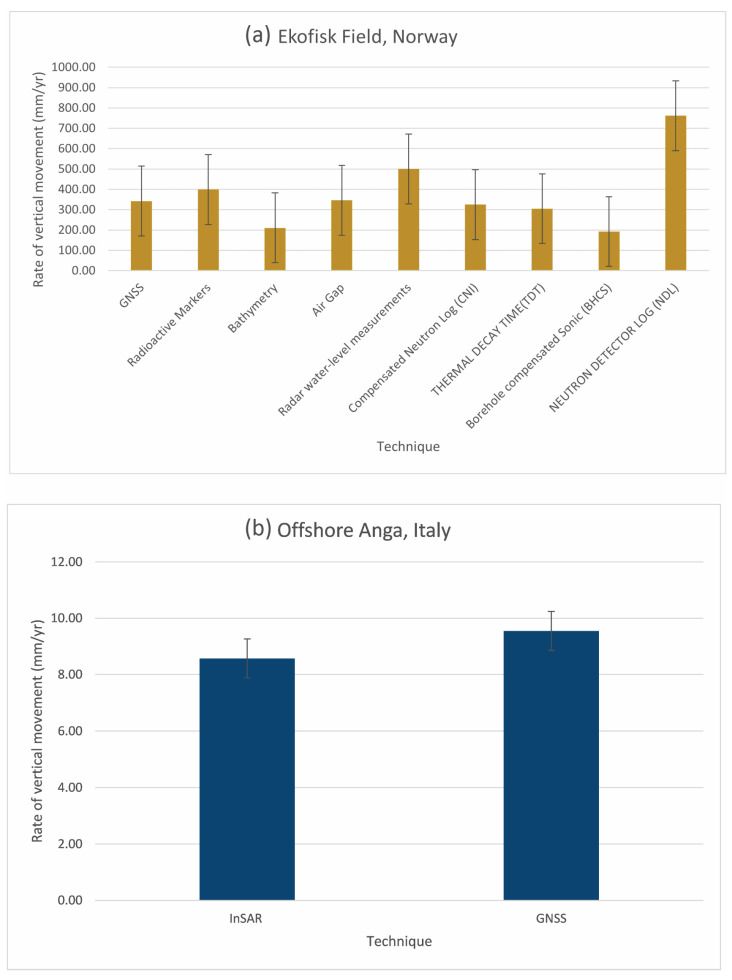
Comparison graphs of the rate of vertical movement (mm/yr) measured using different techniques at (**a**) Ekofisk, Norway and (**b**) Offshore Anga, Italy; bars represent the standard deviation of the data.

Subsidence surveys at Ekofisk have utilized an array of techniques, including bathymetry, air gap measurements, radar water-level measurements, electric log data, the radioactive marker technique (RMT), and global navigation satellite system (GNSS) technology. This eclectic approach has allowed for a detailed comparative analysis, revealing significant variations in the subsidence rates, reflecting the distinct nature of each employed technique. Noteworthy disparities are evident Figure 12a, with numbers ranging from 762 mm/yr derived from neutron logs to 192.5 mm/yr from borehole compensated sonic (BHCS) analyses [33,41,70].

The analysis underscores the distinct capabilities and limitations of each method, with the impact of operational factors like production shutdowns further complicating the subsidence rates. These shutdowns, as highlighted by [70], introduce detectable changes in subsidence patterns, typically peaking within two to four months post-interruption.

Figure 12 facilitates a comparative assessment of different techniques used at two distinct offshore locations: Ekofisk, Norway, and Offshore Anga, Italy. Additionally, it provides insights into the variability within the data through standard deviation analysis. These figures collectively contribute to a comprehensive understanding of subsidence monitoring methodologies.

### 4.2. Anga, Italy

An essential example of a comparative study on the usage of GNSS and InSAR in monitoring platform subsidence that would be addressed in Italy is the Anga case. The Anga platform, located on the coast of the Upper Adriatic Sea, can be taken as a model depicting the multifaceted contribution of both natural and anthropogenic elements to ground settlement.

The study, spanning from 2012 to 2017, by Polcari et al. [20] divulged subsidence rates and corresponding seasonal variances, underscoring the criticality of precise and cross-validated geodetic measurements in monitoring such phenomena. As seen in Figure 12b, the GNSS showed an average subsidence rate of −9.55 mm/year, whereas InSAR, utilizing radar signals from space to create high-resolution images, measured a slightly lower average subsidence rate of −8.57 mm/year. The highest subsidence rate recorded by InSAR reached −21 mm/year, while GNSS data reported a maximum of −17 mm/year, demonstrating a certain degree of variation between the two techniques.

These findings are supported by significant seasonal signals, likely tied to thermal expansion and contraction effects on the platforms, which were similarly constrained in both datasets. Cross-validation procedures further demonstrated strong linear correlations between the diverse time-series data across various sectors and at the offshore platform, with R-squared values ranging from 0.8 to 0.95, showcasing the robustness of the integrated geodetic approaches in subsidence detection.

### 4.3. Valhall Field, Norway

Located in the central graben of the North Sea, the Valhall field is at a water depth of 69 m, encompassing an over-pressured and under-saturated Upper Cretaceous chalk reservoir. This reservoir, lying 290 km offshore and at a depth of 2400 m, [93], exhibited significant sea-floor subsidence, measuring 500 mm just three years after production began.

This subsidence was subsequently measured using pressure gauges. A consistent subsidence rate of 250 mm per year was recorded [52]. The pronounced subsidence rate was attributed to the considerable compaction experienced by the high-porosity chalks upon depletion. Notably, in 2003, a permanent seismic array known as the LoFS was established, encompassing an area of 45 square kilometers. From November 2003 to April 2005, five seismic time-lapse surveys were conducted. The 4D responses detected using this system at Valhall were substantial and were primarily interpreted to result from production-induced reservoir compaction, stress/strain alterations linked to subsidence, and variations in fluid saturation and pressure within the reservoir [48].

Significantly, a groundbreaking, permanent fiber-optic in-well seismic system was developed, representing a global first. This system included five three-component fiber-optic sensors positioned at 13-m intervals along the borehole, utilizing two of the three available optical fibers. Additionally, the installation integrated a pressure/temperature gauge within the reservoir section, occupying the remaining optical fiber. These fiber-optic sensors serve dual purposes, capturing data for both active (4D imaging) and passive (micro-seismic monitoring) applications. A comprehensive analysis of the acquired data revealed high-resolution structural images within a section where surface seismic coverage was compromised due to an overlying gas cloud [48].

## 5. Discussion

For metadata analysis, the Scopus database was employed to assess the most impactful scholarly contributions. Despite the existence of certain articles with a greater number of citations cited within this study, our selection prioritized articles that specifically centered around the domain of offshore subsidence monitoring.

**Figure 13:**
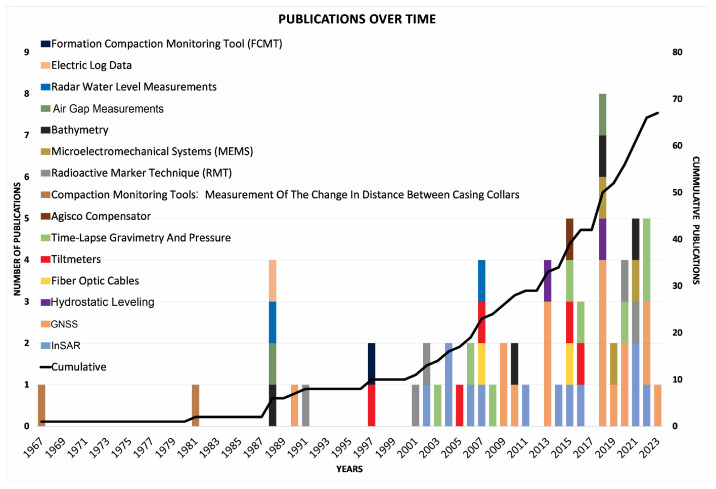
Progression over time of publications dealing with the topic of subsidence monitoring, categorized according to the specific methods used in the studies.

In Figure 13, a chronological representation is presented, illustrating the progression of articles referenced in this study with respect to each respective technique’s evolution. The topic increased importance since the early 2000s, and the GNSS and InSAR exhibit a pronounced surge in research activity during recent years.

**Figure 14:**
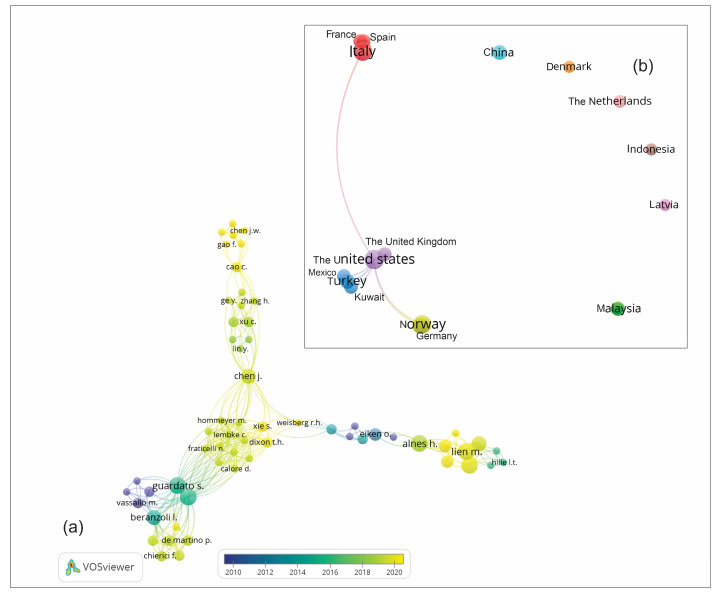
(**a**) Network of collaborating researchers over time, showcasing the evolution and dynamics of research collaboration within the field. (**b**) Highlight on the countries that have contributed the most to research on offshore subsidence monitoring, providing insights into geographical patterns and concentrations of research activity.

A network graph representing the connections among research groups and their temporal evolution grouped also by country (Figure 14) was generated utilizing VOS software (https://www.vosviewer.com/ accessed on 15 August 2023). The data used for this visualization were extracted from the Scopus database.

It is noteworthy that the earliest research stemmed from a few isolated research groups in Europe (i.e., Italy and Norway). Later research involved scientists from the United States and Canada, before a fast spread in the 2010s to other countries worldwide.

**Figure 15:**
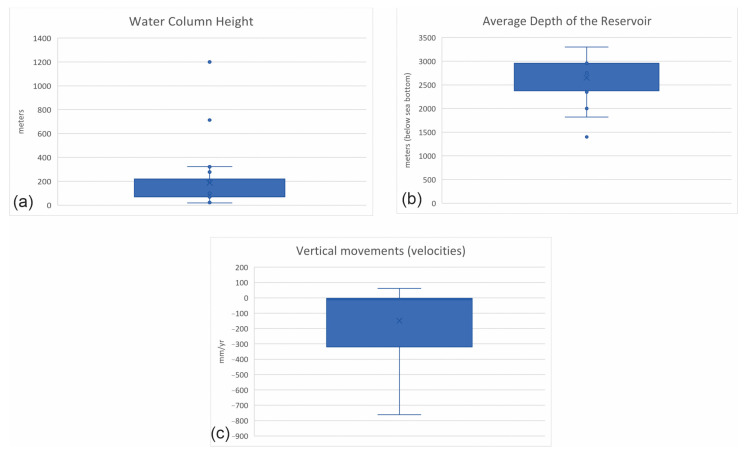
Boxplot graphs illustrate key parameters related to subsidence monitoring. (**a**) Distribution of average water column height measurements, providing insights into variations over time or across different locations. (**b**) Distribution of average reservoir depth measurements, offering an understanding of the reservoir’s characteristics and potential impact on subsidence. (**c**) Distributions of measured vertical velocities, which are crucial indicators of subsidence dynamics.

In Figure 15a, the distributions of some of the parameters characterizing the analyzed case studies are depicted. The water column height, providing insights into the various techniques used, is between 20–70 m on average with a relatively tight distribution. The average depth of the reservoir (Figure 15b), a critical factor in subsidence monitoring, is between 1800–2900 m on average. The velocities of the detected vertical movements (Figure 15c) are in the order of hundreds of millimeters per year, far beyond the accuracy of the techniques, as described in the sections above.

**Figure 16:**
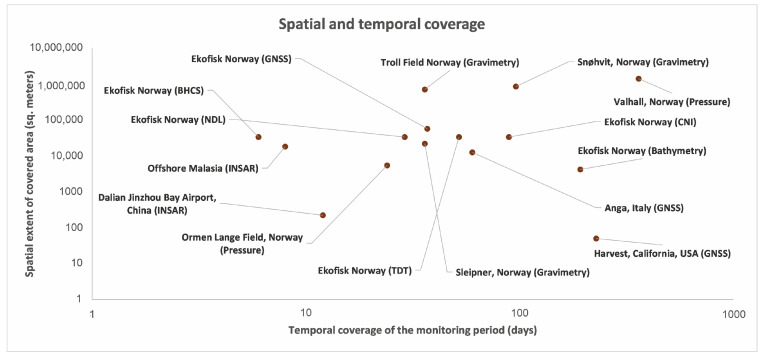
Spatial extent of monitoring for various case studies, depicted alongside the corresponding time periods in days. This visualization provides valuable insights into the duration and coverage of monitoring efforts across different geographical regions and study areas.

Figure 16 illustrates the temporal and spatial scales of the analyzed monitoring campaigns, based on the duration of the data collection and on the spatial extent of the area covered by the surveys. It is evident that a large range of values for both the scales are covered by the analyzed case studies. The spatial extent ranges from a few hundred square meters to tens of square kilometers and the monitoring time window from some weeks to years.

**Figure 17:**
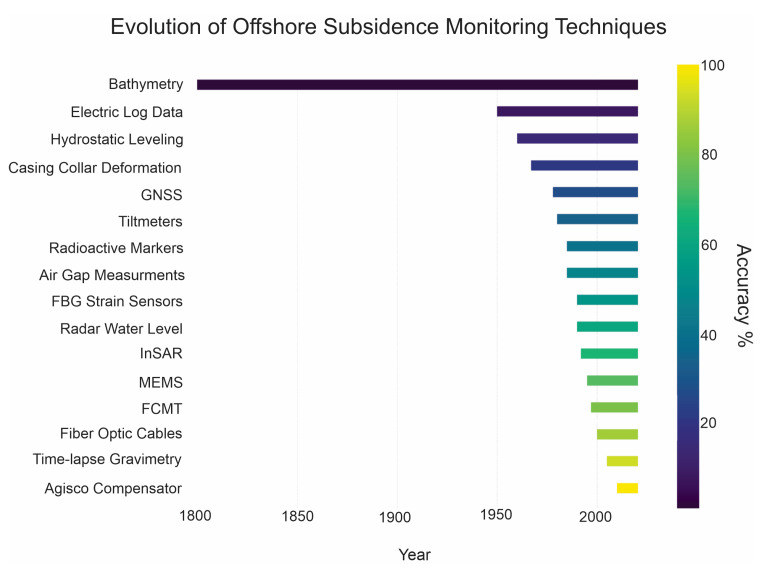
Evolution through time of offshore subsidence monitoring techniques, with their level of accuracy from Table 2. Techniques introduced more recently generally show a higher level of accuracy.

Table 2 and Figure 17 present a comprehensive compilation of the advantages and disadvantages associated with each monitoring technique. While the table covers a range of facets, our primary emphasis centers on evaluating the operational convenience of these technologies in terms of their compatibility with offshore platform activities. We particularly consider their non-intrusive nature and their installation requirements, specifically pertaining to the deployment of equipment on the platform or within subsurface formations. Techniques such as the radioactive marker technique (RMT), formation–compaction monitoring tool (FCMT), electric logs, and casing collar deformation analysis, for instance, necessitate installation within wells or formations.

Our evaluation also factors in spatial and temporal considerations that impact the applicability of these technologies. We account for installation methodologies on the seabed and potential environmental influences, which are notably diverse in the marine environment. It is important to acknowledge that certain complexities render some technologies unsuitable for deployment in deep-sea scenarios. Additionally, data collection from isolated or remote installations may pose challenges, particularly in terms of accessibility for post-observation data retrieval. The discussion also delves into the nature of data collection, whether it involves single-point measurements (as observed in the GNSS and InSAR) and whether these technologies are adaptable for horizontal wells. The complexities surrounding point data are highlighted, especially concerning the study of compartmentalization, an aspect of substantial economic importance in subsidence analysis.

It’s worth noting that remote sensing technologies offer the advantage of multi-temporal monitoring, facilitating the tracking of subsidence over time by analyzing multiple radar images obtained at different instances. This capability enables the identification of trends and changes in subsidence patterns.

## 6. Conclusions

This review has shown the significance of the use of monitoring for subsidence in offshore oil and gas operations. We summarize the most used techniques, emphasizing their advantages as well as limitations. The main conclusions of our research can be summarized as follows:

The spatial coverage and accuracy are highly variable. InSAR and GNSS methods are advantageous because of their extensive coverage and high accuracy, while methods like the RMT or FCMT would not provide wide spatial information, although they can be employed in specific targeted areas with measured values. To decide the most suitable method, a case-by-case approach should be pursued, considering the size of the project as well as its depth and environmental conditions. In addition to the negative effects of subsidence, the utilization of subsidence monitoring and modeling to predict and understand subsidence can provide valuable insights into the compartmentalization of underground reservoirs. The Midgard field serves as an example, as its subsidence levels were lower than predicted in a specific area, prompting a re-analysis of fault seals in the Delta segment. By reinterpreting seismic data and generating various versions of fault interpretations, the study revealed that the eastern portion of the segment may be more isolated than previously assumed. This knowledge can offer useful guidance for optimizing drilling and production strategies in the reservoir while also enabling the identification and mitigation of subsidence-related hazards. The relevance of subsidence monitoring extends beyond the oil and gas industry. For example, subsidence monitoring can track the effects of drought or the over-exploitation of aquifers, indicating changes in groundwater levels. Uplift measurements are also valuable in controlling CO_2_ injection when combined with well pressures. Additionally, subsidence maps can be inverted to reveal variations in rock compressibility and reservoir pressure, helping to identify undrained compartments in oil or gas fields. Finally, the accuracy of reservoir flow and geomechanical models can be enhanced by comparing real subsidence data with model predictions. The combination of different methodologies and the adoption of new methods, including MEMSs and AI, can help improve land subsidence detection. These developments will be beneficial in the areas of prediction modeling, real-time risk analysis, and determining environmental effects of offshore structures so that safer, environmentally friendly activities can be ensured.

## Figures and Tables

**Table 2:** Table of all the techniques along with the advantages and disadvantages.

Technique	Advantages	Limitation
InSAR Accuracy: 1 mm to 10 mm Depth of water column does not affect measurements	Multi-temporal monitoring	Limited vertical accuracy
	All-weather monitoring capability	Not suitable for horizontal wells, as subsidence bowl can be away from the platform
	Repeatability of measurements	Tropospheric distortion and correction are not fully developed
	Extensive coverage	Knowledge of compartmentalization can be restricted, as those data are restricted to a few data points to be spatially interpolated
		Requires line of sight to the target area
GNSS Accuracy: 5 mm to 20 mm Depth of water column does not affect measurements	Precise measurements	Requires clear sky view for optimal performance
	Extensive coverage if installed on multiple platforms	Ionospheric distortions
	Continuous monitoring	Satellite multipath effects
	Remote monitoring	Inaccuracies in satellite orbits
	Non-invasive to other operations	Tropospheric anomalies
	Long-term data collection	Not suitable for horizontal wells
	Integration with other data (GNSS + inclinometers; pressure sensors)	Knowledge of compartmentalization can be restricted, as those data are restricted to a few data points to be spatially interpolated
Hydrostatic leveling Accuracy: 1 mm to 10 mm Shallow waters	High precision	Limited resolution
	Direct measurement	Limited coverage
	Stable and robust: hydrostatic leveling is less susceptible to atmospheric conditions	Labor intensive: the setup and operation of hydrostatic leveling systems can be labor intensive, requiring frequent site visits for measurements
	Continuous monitoring	Prone to long-term drift, requiring periodic recalibration
		Single-point measurements
Fiber optic cables Accuracy: 1 mm to 5 mm Deep sea depending on cable design	High sensitivity	Installation complexity
	Continuous monitoring	cost
	Distributed sensing	Limited coverage
	Non-intrusive	Data transfer and storage challenges due to large volumes of data
	Multipurpose: can monitor strain, temperature, and other environmental parameters	Calibration and validation: regular calibration and validation of fiber optic sensors are necessary
(FBG) strain sensors Accuracy: 1 mm to 5 mm Deep-sea environments depending on cable design	High sensitivity	Initial cost
	Distributed FBG sensors can be positioned along a single optical fiber, enabling distributed sensing over a large area	Limited absolute measurements: the sensors measure strain relative to their initial state, making them more suitable for detecting changes rather than providing absolute subsidence measurements
	Real-time monitoring	Complexity of interpretation
		Requires sophisticated data analysis techniques
Tiltmeters Accuracy: 0.01 to 1 arcsecond Deep sea depending on design	High sensitivity	Limited coverage
	Direct measurement	Calibration and adjustment
	Variety of applications beyond subsidence monitoring, such as structural health monitoring and landslide detection	Limited range
		Installation complexity
Agisco compensator Accuracy: 10 mm to 100 mm Shallow to moderate depths	High precision	Installation and calibration
	Directly measures subsidence	Limited vertical range
	Can provide real-time or near-real-time data on subsidence events	Regular maintenance and occasional repairs may be required
	Long-term monitoring	Limited coverage
	Can be integrated with other monitoring techniques	Regular maintenance and occasional repairs may be required to ensure the instrument’s accuracy
Time-lapse gravimetry and pressure accuracy: 10 mm to 100 mm Deep-sea use depending on equipment design	Direct measurement	Calibration challenges
	Long-range monitoring	Deployment challenges
	Non-intrusive	Limited accessibility: maintenance and repair
Casing collar deformation analysis Accuracy: 1 mm to 10 mm Shallow to moderate depths	Direct measurement	Limited measurement locations
	Cost effective	Restricted vertical range
	Long-term monitoring	Dependency on casing collars: accurate measurements depend on the integrity and stability of the casing collars; any shifts or movements in the collars can affect data accuracy
		Lack of continuous data
		Requires detailed baseline data for effective comparison
Radioactive marker technique (RMT) Accuracy: 1 mm to 5 mm Shallow to moderate depths	Direct measurement	Radiation hazards
	High precision	Regulatory approval
	Long-term monitoring	Limited vertical range
	Continuous monitoring	Data interpretation complexity
	Comprehensive coverage: RMT markers can be placed at multiple depths	Marker installation requires specialized equipment and procedures
		Specialized safety protocols required to handle radioactive materials
		Expensive
Microelectromechanical systems (MEMSs) Accuracy: 1 mm to 5 mm Deep-sea environments depending on sensor design	High precision	Calibration and stability: requires regular calibration and can be prone to stability issues over time
	Compact size	Limited range
	Real-time monitoring	Sensor drift
	Cost effective	Sensor lifetime
	Ease of installation	Data processing complexity
	Durability: MEMS sensors are designed to withstand harsh environmental conditions, including exposure to moisture and corrosive elements	Data transmission reliability: wireless communication might be affected by interference, signal attenuation, or communication range limitations
	Multiparameter monitoring	
	Wireless communication	
	Low power consumption	
	Versatile applications: capable of monitoring a wide range of physical parameters	Calibration and stability: requires regular calibration and can be prone to stability issues over time
Bathymetry Accuracy: 1 mm to 1cm Shallow and deep sea	Comprehensive mapping	Limited vertical precision
	Non-intrusive	Interference from structures such as pipelines
	Large area coverage	Depth limitations
	High accuracy	Lack of real-time monitoring
Air gap measurements Accuracy: 10 mm to 100 mm Unaffected by water column height	Direct vertical measurements	Dependent on tidal variations
	Simple and cost effective	Limited range, especially for larger subsidence events
	Real-Time Monitoring	Continuous monitoring challenges
	Early warning indicators of potential subsidence or structural issues	Environmental factors like wind and waves can introduce noise into air gap measurements
		Can be influenced by sea state and vessel motion
Radar water-level measurements Accuracy: 1 cm to 20 cm Shallow environments	Non-contact measurement physically with the water	Environmental factors like wind and waves can introduce noise into air gap measurements
	Continuous monitoring	Tidal fluctuations can impact water level measurements
	Remote sensing	Radar signals can be affected by interference from other structures, equipment, or vessels in the vicinity
	Large coverage area	Cost
Electric log data Accuracy: 10 mm to 100 mm Depends on the well	Available historical data	Limited to well locations
	Multi-well monitoring	Costly and time consuming
	Direct measurement of subsurface changes	Invasive process, as obtaining data requires accessing and instrumenting wells, which might interfere with ongoing operations
	High vertical resolution	Dependent on well conditions and logging technology
		Limited temporal resolution
Formation–compaction monitoring tool (FCMT) Accuracy: 1 mm to 10 mm From shallow to relatively deep waters, up to around 3000 m	Direct measurement of compaction	Localized monitoring
	High precision	Installation and data retrieval
	Long-term monitoring	Dependency on well access
	Specific to reservoir conditions: FCMT can be customized to suit the specific geological and reservoir conditions	Limited data points
	Customizable to specific geological settings	

## Data Availability

No new data were created or analyzed in this study. Data sharing is not applicable to this article.
